# The common morphospecies *Cypridopsis vidua* (O.F. Müller, 1776) (Crustacea, Ostracoda) is not an obligate parthenogen

**DOI:** 10.26496/bjz.2023.107

**Published:** 2023-01-19

**Authors:** Koen Martens, Michael Shribak, Irina Arkhipova, Isa Schön

**Affiliations:** 1 Royal Belgian Institute of Natural Sciences, Freshwater Biology, Vautierstraat 29, Brussels 1000, Belgium.; 2 Ghent University, Department of Biology, K.L. Ledeganckstraat 35, 9000 Ghent, Belgium.; 3 Marine Biological Laboratory, Woods Hole MA, USA.; 4 Centre of Environmental Sciences (CMK), University of Hasselt, 3590 Diepenbeek, Belgium.

**Keywords:** Mixed reproduction, male morphology, copula, insemination, polychromatic polarization microscopy

## Abstract

The common non-marine ostracod *Cypridopsis vidua* (O.F. Müller, 1776) is used as a proxy in various biological disciplines, such as (palaeo-)ecology, evolutionary biology, ecotoxicology and parasitology. This morphospecies was considered to be an obligate parthenogen. We report on the discovery of the first population of *C. vidua* with males from Woods Hole (MA, USA) and determine that it is a population with mixed reproduction. We describe the morphology of the males and of the sexual and asexual females. We illustrate a copula of a male and a sexual female as well insemination in a sexual female, showing that males are functional. Therefore, *Cypridopsis vidua* is a morphospecies with mixed reproduction, not a full apomictic parthenogen. We use, for the first time, polychromatic polarization microscope technology to illustrate soft parts of ostracods. In addition, we compare the sexual species *C. bisexualis*, *C. okeechobei*, *C. howei* and *C. schwartzi* and conclude that these species, especially the latter three, are morphologically very close to *C. vidua*.

## Introduction

Non-marine ostracods, small, bivalved crustaceans, are a common part of many types of aquatic ecosystems. Currently, approximately 2330 species and 270 genera of living non-marine ostracods are known worldwide (Meisch *et al*. 2019). Ostracods are excellent model systems for evolutionary research, as they are the living arthropod group with the most extensive fossil record, enabling studies of evolutionary pathways in real-time frames (Martens & Horne 2000). In addition, there is a large incidence of parthenogenetic reproduction and both sexual and asexual reproduction can occur in the same species. Such species are generally referred to as having mixed reproduction and comprise asexual females, males and sexual females. This is especially so in species of the family Cyprididae Baird, 1845, with more than 1000 recognised species comprising ca 43% of the total specific non-marine ostracod diversity (Meisch *et al*. 2019).

Parthenogenesis in ostracods is very different from the commonly known cyclic parthenogenesis, such as in cladocerans, where populations experience several generations of asexual reproduction in favourable seasons, while the last cycle(s) before the unfavourable season (winter, dry season) are sexual with males and sexual females. This generation then produces ephippia to survive the unfavourable season (Decaestecker *et al*. 2009).

Sex in non-marine ostracods, especially in Cyprididae, is mostly determined by genetic mechanisms, with males being the heterogametic gender (Schön *et al*. 1998; Meirmans 2009). However, other factors such as cytoplasmatic micro-organisms (e.g., *Cardinium* infections - Schön *et al*. 2018; Schön & Martens 2020) could also play a role in sex determination.

There was a tendency amongst some ostracod workers in the mid-20^th^ century to consider sexual populations of otherwise asexual species as different nominal species. The best-known example in North America is that of sexual populations of *Limnocythere inopinata* (Baird, 1843) which were commonly referred to as *Limnocythere sappaensis* Staplin, 1963. Meanwhile, genetic work has shown that sexual and asexual strains of the same species cluster together in molecular trees (e.g., Bode *et al*. 2010 on *Eucypris virens* (Jurine, 1820)). These insights have led Meisch (2000) to formally sink *Limnocythere sappaensis* into the synonymy with *L. inopinata*. We will consider sexual and asexual populations as conspecific in the present paper.

Mixed populations with asexual and sexual females plus males can lead to hybridisation between males and asexual females, giving rise to polyploid (asexual) strains (Adolfsson *et al*. 2009; Symonová *et al*. 2018). The combination of these (and other) factors can, over time, give rise to species clusters, with several cryptic genetic species existing within the traditional morphospecies. A case in point is the cypridid ostracod species *Eucypris virens* in which close to 40 (cryptic) genetic species were found in Europe (Bode *et al*. 2010). Some of these genetic species appear to be fully asexual, others are mixed, few are fully sexual. It is assumed that such entities can be evolutionarily dynamic, with some genetic species going extinct, while others arise *de novo*, for example by intra-specific hybridisation (Bode *et al*. 2010).

*Cypridopsis vidua* (O.F. Müller, 1776) is one of the most common non-marine ostracod species in the Holarctic, but has also been reported from all other continents, except Antarctica (Meisch 2000; Meisch *et al*. 2019). It has been used as a proxy in various biological disciplines, such as (palaeo-)ecology, evolutionary biology, ecotoxicology and parasitology (see below). Only fully asexual populations have thus far been found so that Havel & Hebert (1989) considered it an obligate apomictic parthenogen, this in spite of the fact that the species is quite variable in size, shape and colouration of its carapace and many slightly deviant populations have been described as different species. Meisch *et al*. (2019) cited no less than 19 synonyms. A molecular study of the genetic diversity of this common morphospecies is underway and will be presented elsewhere (Cours *et al*. in prep.).

Here, we report on the first discovery of a mixed population of *Cypridopsis vidua* from Woods Hole (MA, USA) and describe aspects of the morphology of the males as well as of sexual and of asexual females. We also describe a copula of a male and a sexual female and a case of *in situ* fertilization in a sexual female of *C. vidua*. For the first time, we use polychromatic polarization microscopy (Shribak 2014, 2015) to illustrate soft parts of ostracods.

## Material and methods

### Study area and material

Samples were collected from an algal freshwater pool on Gardiner Rd, surface ca 10 × 20 m, near the Marine Biological Laboratory (MBL) in Woods Hole, MA, USA (see Appendix, Fig. S1A) on August 12^th^, 2019 by KM and IS using a hand net with mesh size of 160 μm. Approximate coordinates: 41°528572 N, 70°672693 W. The population of *C. vidua* in this pool consisted of asexual females, males and sexual females. At the time of collecting, the pond was filled and covered by green algae (Fig. S1A), which were, one week later, manually largely cleared by municipal services (Fig. S1B). In 2022, the pond had changed to a different ecological state, with many submerged and emergent macrophytes, but still with patches of submerged algae (Fig. S1C–D). The mixed population of males, sexual and asexual females of *Cypridopsis vidua* had persisted in high densities, with dozens to hundreds of specimens in each net sample.

### Methods

Samples were fixed in 99% ethanol and ostracods were sorted under a WILD M10 binocular microscope and dissected with valves stored dry in micropaleontological slides and with soft parts dissected with tungsten needles in glycerine in sealed slides. Drawings of soft parts were made with a *camera lucida* with a compound microscope (Olympus, BX51 at RBINS, Brussels).

Hemipenes and prehensile palps were also illustrated using a novel microscopic technique. The polychromatic polarization microscope is a unique instrument that uses polarization interference colours to show details of tissues that would otherwise be invisible. The polychromatic polarization microscope was invented by Michael Shribak (Shribak 2014, 2015) at the Marine Biological Laboratory (MBL, Woods Hole, MA, USA) to visualize even weak birefringent and small structures in colours. Usually, Newton colours appear in microscopic pictures only if one of the two white light beams is retarded relative to the other by 400 nm to 2000 nm (Shribak 2014, 2015; Rajabi *et al*. 2022). Structures with smaller retardance as they are typical for (small) ostracods then appear as grey images. In the polscope, ector interference of polarized light creates full spectrum colours at retardance of several nanometers, whereby the hue is determined by orientation of the birefringent structure. Previously colourless birefringent images of organelles, cells, and tissues thus become vividly coloured (Shribak 2015). The “polscope” set-up here consisted of a microscope Olympus IX81, with objective lens magnification 20 × and total magnification 20 × 16, and a camera: colour CCD camera Olympus DP73.

Valves were illustrated and measured using Scanning Electron Microscopy (SEM; Fei Qanta 200 ESEM, Royal Belgian Institute of Natural Sciences, Brussels, Belgium). Higher taxonomy of the Ostracoda follows the synopses by Horne *et al*. (2002) and by Meisch *et al*. (2019). The picture of copula of the male and the sexual female was taken with a binocular microscope at MBL (Woods Hole, MA, USA) by Mr Chris Dix, and was augmented using Adobe Photoshop Elements.

Reference material was deposited in the Ostracod Collection of the Royal Belgian Institute of Natural Sciences, Brussels, Belgium (INV – numbers).

## Results

### Taxonomic account

Class Ostracoda Latreille, 1802

Subclass Podocopa Sars, 1866

Order Podocopida Sars, 1866

Suborder Cypridocopina Baird, 1845

Superfamily Cypridoidea Baird, 1845

Family Cyprididae Baird, 1845

Subfamily Cypridopsinae Kaufmann, 1900

Tribe Cypridopsini Kaufmann, 1900

Genus ***Cypridopsis*** Brady, 1867

**Type species** (by original designation). *Cypris vidua* O.F. Müller, 1776.

**Other species**. See Meisch *et al*. (2019).

***Cypridopsis vidua*** (O.F. Müller, 1776)

Figs 1–7

**Synonymies**. Meisch *et al*. (2019) listed 19 synonymies of this species.

**Extended diagnosis** (adapted from Meisch 2000)

Cp ovate in both lateral and dorsal views, usually with four light to dark green transverse bands on yellowish background, but with a remarkable variability in shape, size and external ornamentation. LV slightly longer than RV, overlapping RV at least ventrally and anteriorly, both valves about equally long at the posterior end. CpD with anterior end bluntly pointed, posteriorly more rounded. L of females 0.4–0.7 mm (usually 0.5–0.6 mm), i.e., as typical of cypridopsines.

Posteroventral marginal zone of LV with the double folded inner list, not running parallel to the valve margin. In RV, this list running parallel to valve margin and to selvage. Anterior external marginal zone of RV with a row of ca 15–20 tiny pustules.

A2 with natatory setae extending beyond tips of terminal claws with ca ¼ of their total length. Md-palp with beta-seta stout and hirsute. Mx_1_-palp with terminal segment cylindrical, longer than broad; teeth bristles of 3^rd^ masticatory lobe smooth. Respiratory plate of T_1_ usually with 5 filaments (rarely 4 or 3). Prehensile palps of T1 in males slightly asymmetrical, first segment of Lpp narrower, distal segment broader than in Rpp. T2 short and strongly developed; seta d_l_ missing, d_2_ well-developed; terminal claw heavily built; seta h_3_ tiny or absent. T3 without special characters. CR reduced to a short triangular base a long terminal flagellum and a short subapical seta in females, absent in males.

Hemipenes with a broadly rounded ms, produced towards the ventral side; ls narrower, distally rounded and subapically with a long ventral finger.

#### Material used

USA – **Woods Hole** (Massachusetts) • numerous males and females, the latter both sexual and asexual, roadside algal pond at Gardiner Rd, 41, 41°528572 N, 70°672693 W, August 12^th^, 2019, I. Schön & K. Martens leg., INV.190336–190344. See description of pond above.

#### Descriptions of *C. vidua* from Woods Hole population

##### Male

**Figure 1 –:**
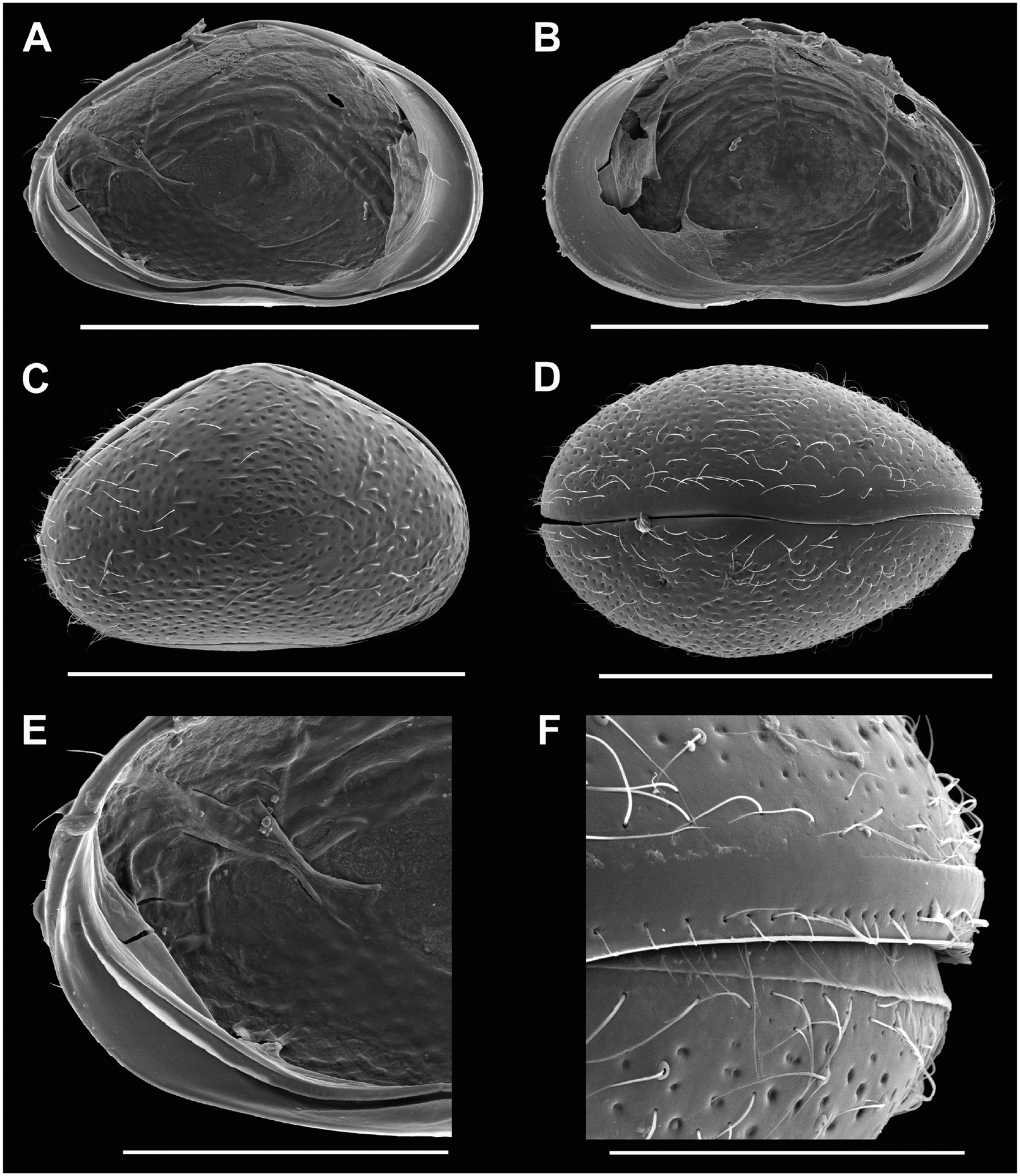
*Cypridopsis vidua*, ♂. **A**. LVi (INV.190336). **B**. RVi (INV.190336). **C**. CpRL (INV.190341/2). **D**. CpD (INV.190341/4). **E**. LVi, detail of posterior part (INV.190336). **F**. CpD, detail anterior (INV.190341/4). Scale bars: A–D = 500 μm; E = 200 μm; F = 100 μm.

Cp smaller than those of females (sexual and asexual); CpRL (Fig. 1C) with greatest height situated well-before the middle, anterior margins more broadly rounded than posterior one. CpD (Fig. 1D, F) narrower than in female, with greatest width situated slightly behind the middle. External ornamentation (Fig. 1F) consisting of clear pits and long and stiff setae.

LVi (Fig. 1A, E) with posterior double inner list not running parallel to valve margin. The latter running close to valve margin along ventral side, barely reaching to anterior margin; the latter with a submarginal (but parallel) selvage.

RVi (Fig. 1B) with anterior submarginal selvage, posteriorly with selvage inwardly displaced, not parallel to valve margin in posteroventral corner.

Testical tubes running in a bunch from anterior to posterior side of valves and back, both bunches running above the central transversal muscles (Fig. 1A–B).

Morphology of somatic limbs (A1, Md, Mx1, T2, T3) as typical of the species. Sexual dimorphisms present in limbs A2 and T1, as well as in hemipenes, Zenker organ and CR.

**Figure 2 –:**
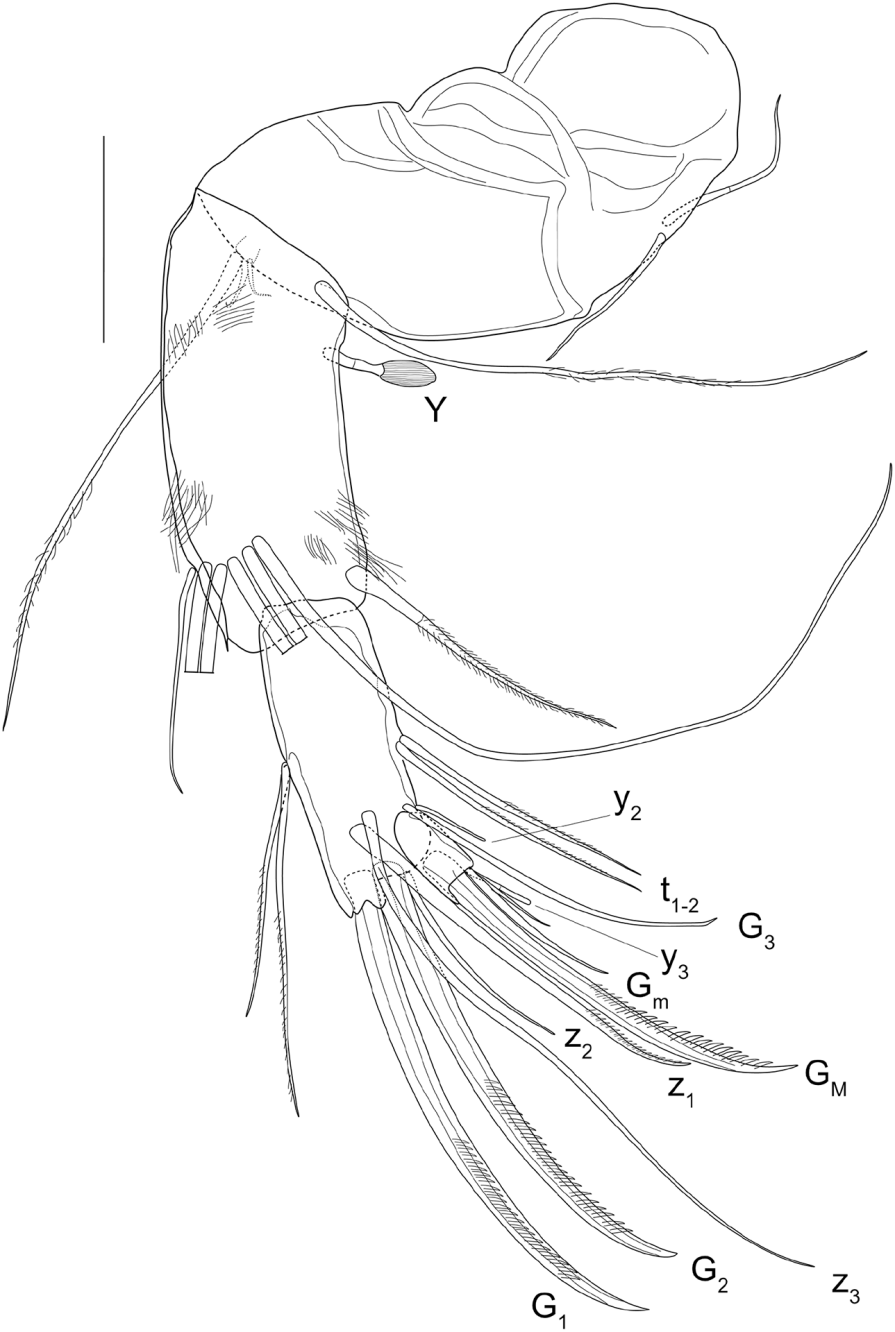
*Cypridopsis vidua*, ♂, A2 (INV.190336). Scale bar: 50 μm.

A2 (Fig. 2) with protopodite, exopodite and three-segmented endopodite. Protopodite ventrally with three setae: two unequal but short basal setae, one long apical seta reaching beyond the tip of the first endopodal segment. Exopodite reduced to a small plate with one long seta (reaching beyond the tip of the first endopodal segment) and two sub-equal short setae. First endopodal segment ventrally with short aestethasc Y (less than ⅓ of the length of this segment), one long hirsute ventral seta (not reaching beyond the tip of the second endopodal segment), five natatory setae, reaching beyond the tip of the end claws, and one short accompanying seta, about ½ of the length of the second endopodal segment. Second endopodal segment with two unequal but long dorsal setae, two long hirsute ventral setae t_1–2_; apically with two large (G_1_, G_2_) and one short (z_1_) claws and three setae (G_3_, z_2_, z_3_. Terminal segment with two claws, one large (G_M_) and one short (G_m_) and one aesthetasc y_3_ with accompanying seta (longer than the aesthetasc). Seta g absent.

**Figure 3 –:**
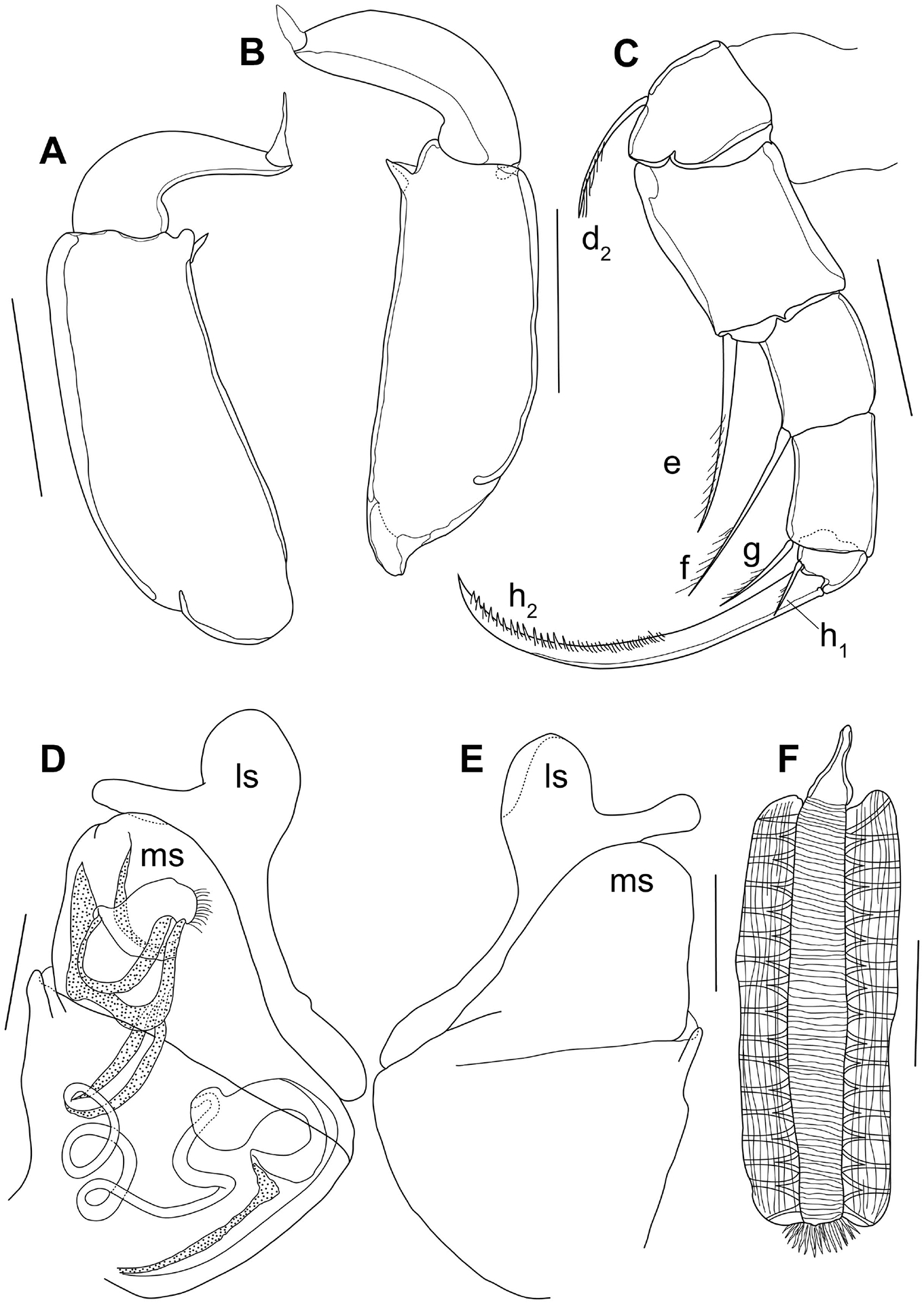
*Cypridopsis vidua*, ♂. **A**. Rpp (INV.190344). **B**. Lpp (INV.190344). **C**. T2 (INV.190344). **D–E**. Hp (INV.190344). **F**. Zenker organ (INV.190344).

**Figure 4 –:**
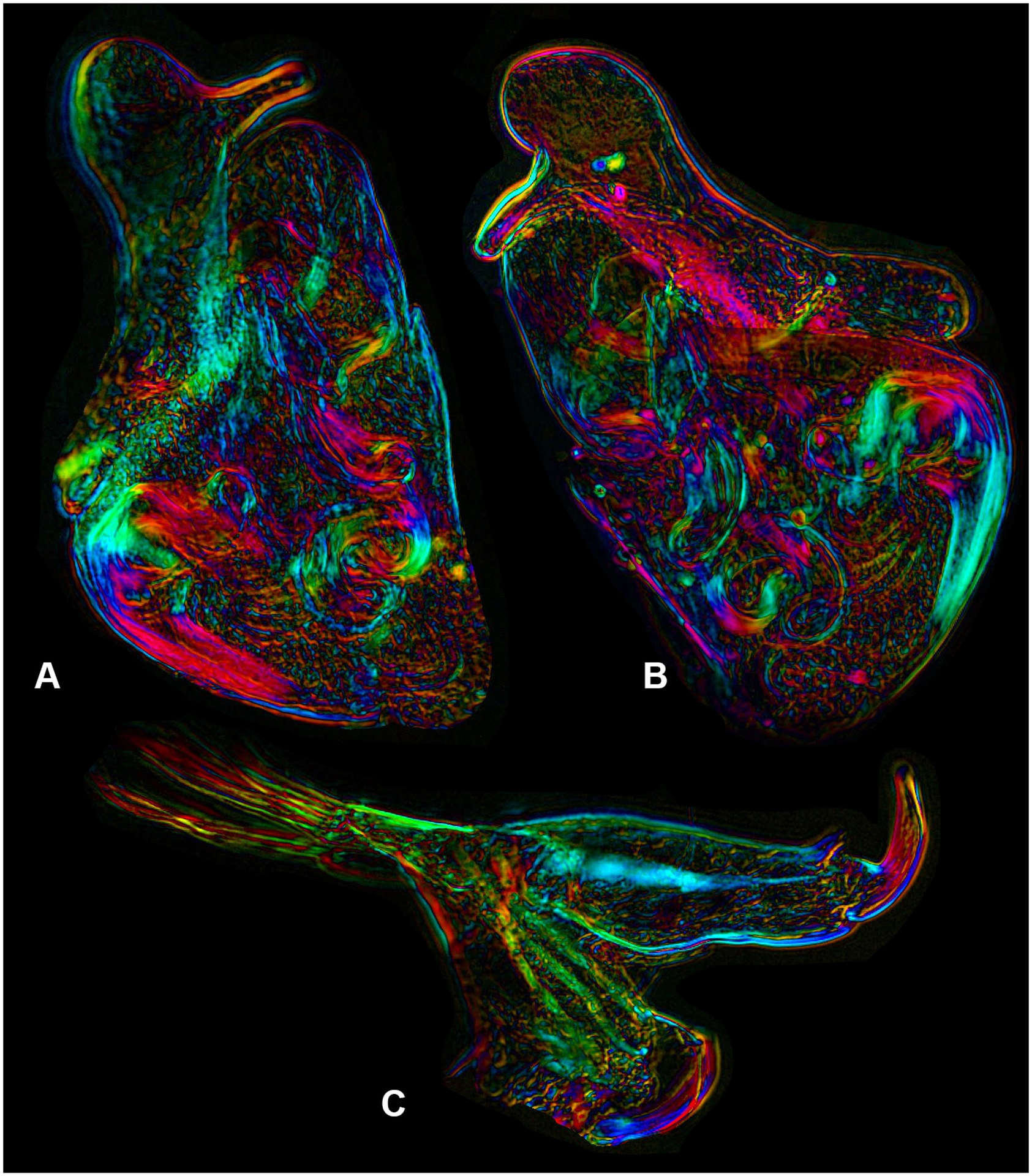
*Cypridopsis vidua*, ♂, (INV.190344). **A–B**. Hemipenes. **C**. Right T1, showing prehensile palp (distal sensory organ folded). All with polychromatic polarization (polscope) microscopy.

Prehensile palps of T1 slightly asymmetrical. Rpp (Figs 3A, 4C) with elongated first segment with subparallel sides, subapically with two small, unequal sensory organs; second segment sickle-shaped, distally tapering, with long distal sensory organ. Lpp (Fig. 3B) with slightly narrower first segment, also subapically with two small sensory organs; second segment broader and less tapering distally.

T2 (Fig. 3C) with seta d1 missing and seta d2 well-developed, as typical of the genus. End claw more robust than in females.

Hemipenes (Figs 3D–E, 4A–B) slightly asymmetrical. Both hemipenes with a large body, distally with a broadly rounded ms, produced towards the ventral side; ls narrower, distally rounded and subapically with a long ventral finger, dorso-proximally with an extended lobe surpassing ms. In one Hp (Fig. 3D), this finger on ls with parallel sides and distally narrowly rounded, touching the edge of the ms. In the second Hp (Fig. 3E), this finger distally swollen and running distant from the edge of the ms. Internally as typical of the Cypridopsinae, with a stout labyrinth, post-labyrinthal spermiduct with two full coils and an additional half coil ending in a stout, cup-like bursa-copulatrix. Internal anatomy especially well-visible in the polychromatic polarization microscope illustrations, with interference colours more clearly showing the post-labyrinthal loops (Fig. 4A–B).

Zenker organ (Fig. 3F) stout and compact, with parallel sides and ca 17 spinous whorls in fully mature males.

CR absent in the male, as typical of the Cypridopsinae.

###### Remark.

The testical tubes and the Zenker organ are very difficult to see in transparency through the carapaces in this species, which makes it easy to miss male specimens in populations which are generally assumed to be all-female. It is quite possible that males have been missed in sexual / mixed populations in the past. We only became aware of the fact that this population contained males, when discovering abundant sperm in a dissection of a female.

##### Female

**Figure 5 –:**
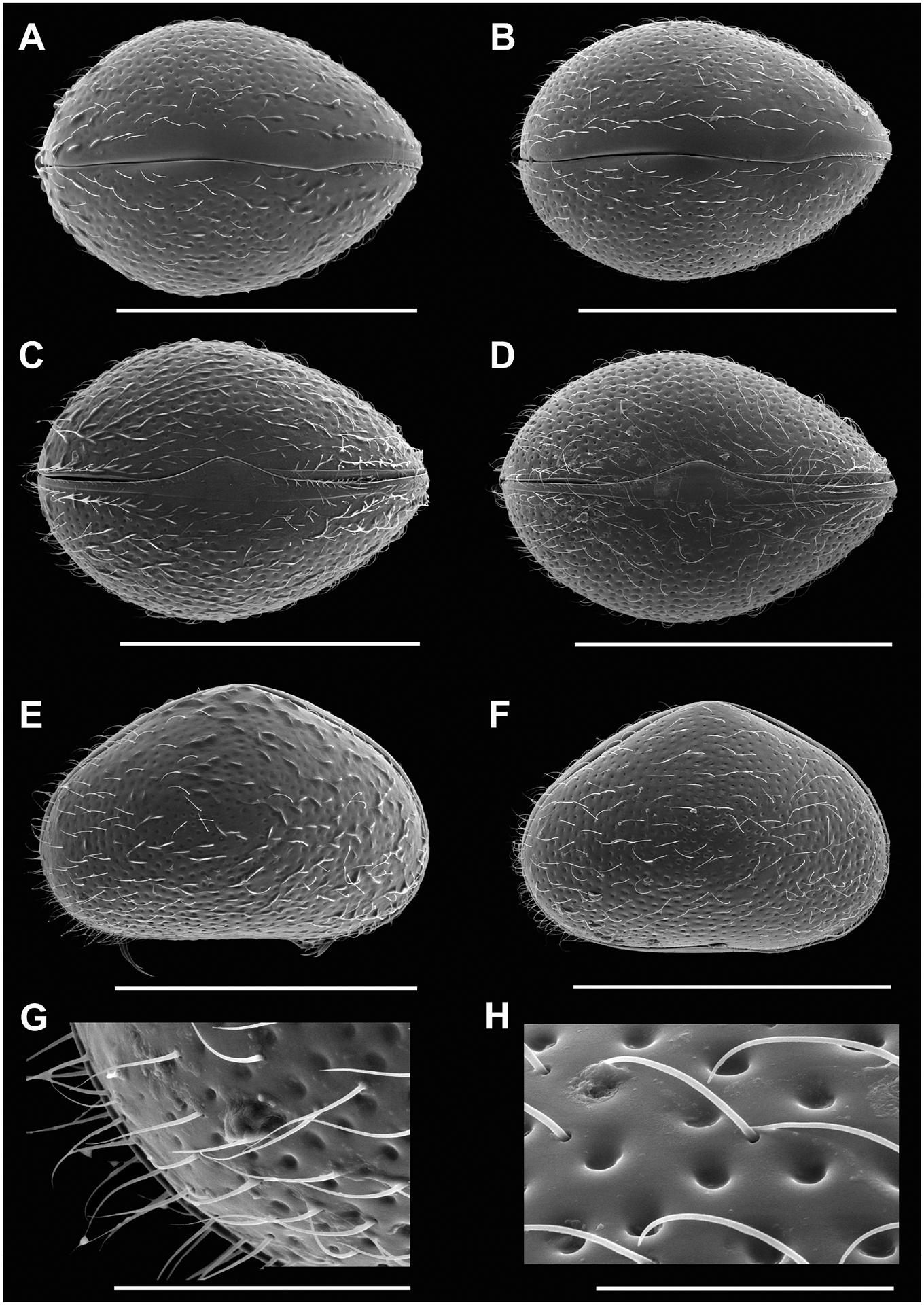
*Cypridopsis vidua*, ♀. **A**. CpD (INV.190338/1). **B**. CpD (INV.190339/1). **C**. CpV (INV.190338/3). **D**. CpV. (INV.190340). **E**. CpRL INV.190338/2). **F**. CpRL (INV.190339/2). **G**. CpRL, detail of carapace surface (INV.190338/2). **H**. CpV, detail of carapace surface (INV.190340). A, C, E, G = asexual; B, D, F, H = sexual. Scale bars: A–F = 500 μm; G = 100 μm; H = 50 μm.

Females assumed to be sexual in the present population (Fig. 5B, D, F – with sperm) were in general slightly smaller than asexual ones (Fig. 5A, C, E – without sperm) and had less pronounced external ornamentation. The most useful distinguishing characters were that asexual females were generally larger and with a more deeply coloured green Cp, while sexual females were smaller, more yellowish of colour (as were the males), but especially that their CpRL was slightly more highly arched (compare Fig. 5E and 5F).

##### Measurements

See Table 1.

#### Insemination

**Figure 6 –:**
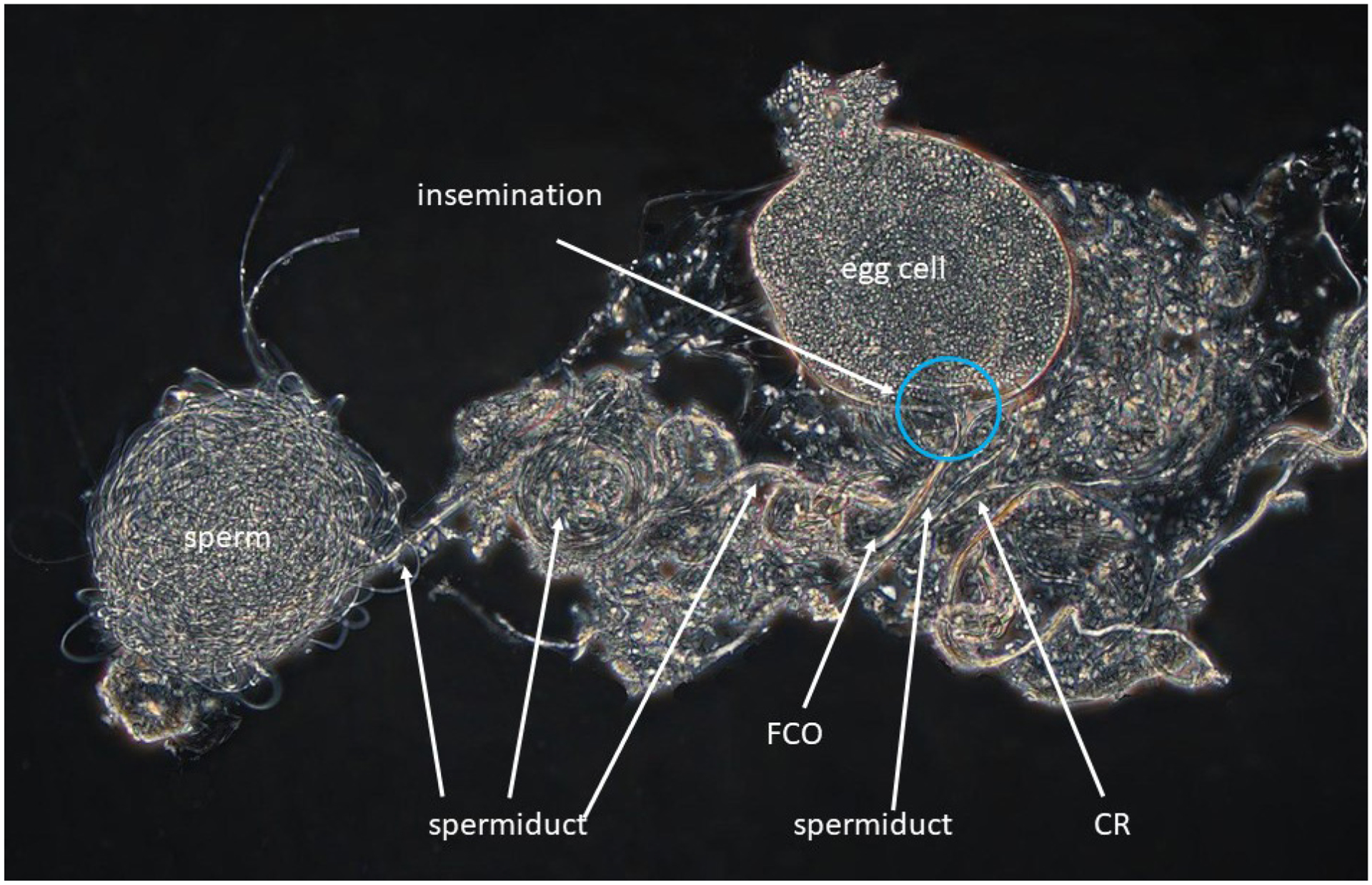
*Cypridopsis vidua*, ♀, sexual (INV.190337). Posterior part of female body, with insemination in progress (no scale). The image was taken with combined phase contrast/polychromatic polarization.

A polychromatic polarization microscopy image of one side of posterior part of a female body (Fig. 6) is showing a mass of sperm on the left (spermatheca not visible), subsequently entering in a spermiduct and subsequent spermiductal coils towards FCO, where insemination in a large egg cell is clearly visible. Also visible are female CR, situated next to the female reproductive organ.

#### Copula

**Figure 7 –:**
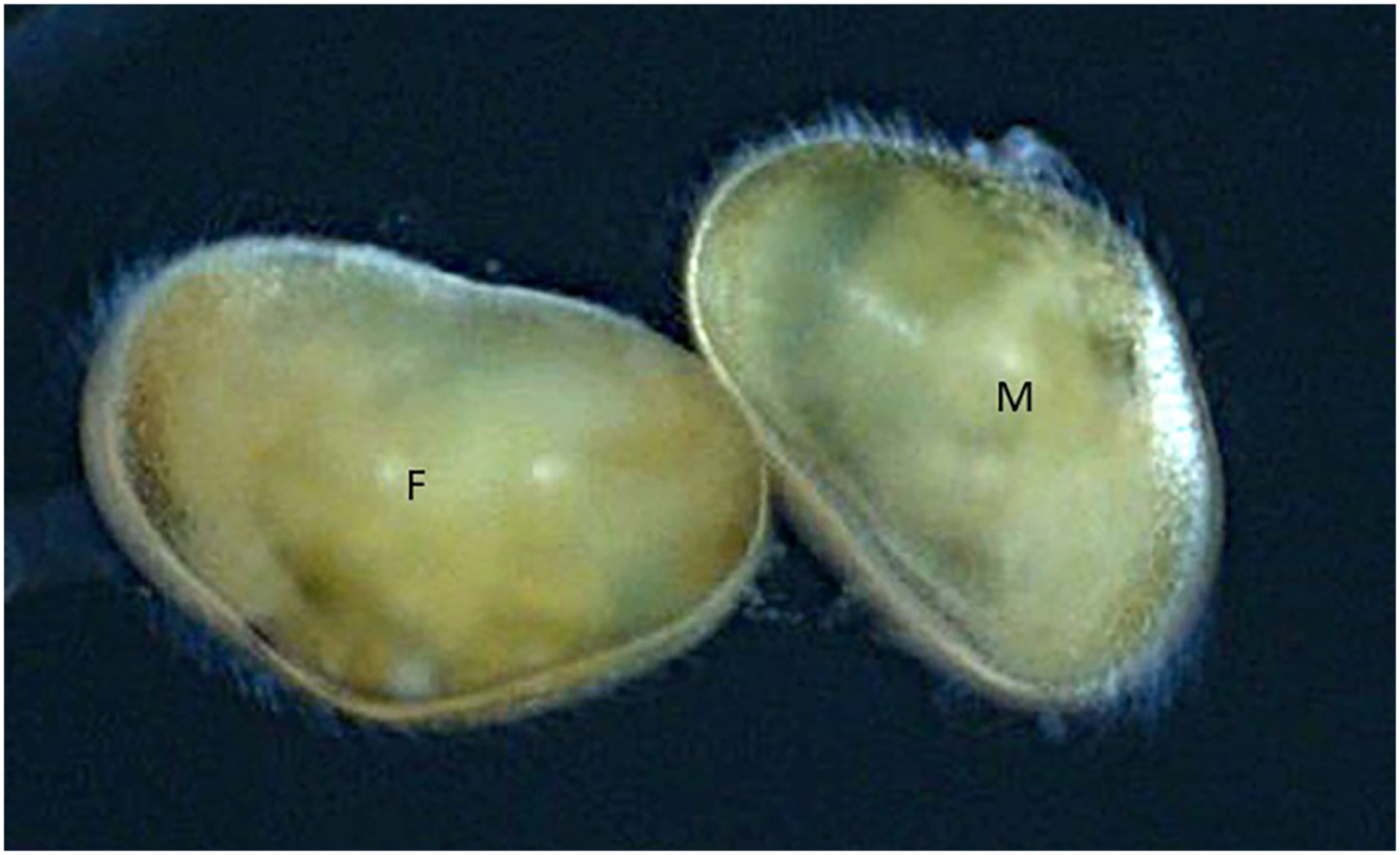
*Cypridopsis vidua*, copula of male (right) and sexual female (left) (no scale).

One male and female were preserved in copulation position (Fig. 7). The present position corresponds to the position “C” in the scheme of Cohen & Morin (1990), which seems quite common in Cyprididae.

## Discussion

### *Cypridopsis vidua* as a model species in biology

*Cypridopsis vidua* is, together with *Cypria ophthalmica* (Jurine, 1820) and *Cyclocypris ovum* (Jurine, 1820), one of the most common species in the Holarctic (Meisch 2000). It occurs in many species inventories, both in recent communities (Meisch 2000) and in fossil assemblages (Griffiths 1995). The species is commonly used in evolutionary studies (Havel & Hebert 1989; Cywinska & Hebert 2002; Schön *et al*. 2019) and especially in ecological assessments, such as its adaptive responses to different types of habitats (Roca & Danielopol 1991; Roca *et al*. 1993) and in behavioural studies (Uiblein *et al*. 1994; Hunt *et al*. 2007), to name only a few. *Cypridopsis vidua* was developed as a proxy for palaeo-temperature reconstructions using Mg / Ca ratios in the valves (Palacios-Fest & Dettman 2001) and as a proxy for sensitivity to radioactivity (Chen *et al*. 2022). *Cypridopsis vidua* is also an intermediate host of an eoacanthocephalan parasite species which has fish as its final host (Lourenço *et al*. 2018).

The above are just a few examples to show that the ostracod species *Cypridopsis vidua* is used as a model or test organism in a variety of biological disciplines. It is therefore more than of academic importance only to determine if this species is just a single apomictically reproducing lineage (Havel & Hebert 1989) or if it is a complex of sexual and asexual lineages through which high morphological and genetic diversity can be maintained.

### The morphospecies *Cypridopsis vidua* is not an exclusive parthenogen

For a long time, it was thought that *Cypridopsis vidua* was an exclusively apomictically reproducing parthenogenetic species (Havel & Hebert 1989), in spite of the fact that it is a hypervariable one, both morphologically (Meisch 2000) and genetically (Cywinska & Hebert 2002). In general, long-term asexual species are thought to be morphologically and genetically uniform, as is the case in the ostracod *Darwinula stevensoni* (Brady & Robertson, 1870) (morphologically: Rossetti & Martens 1996, genetically: Schön *et al*. 1998). High diversity, such as in *C. vidua*, but also in *Eucypris virens* (Jurine, 1820), *Heterocypris incongruens* (Ramdohr, 1808) and other cypridinid ostracod species, is often linked to mixed reproduction (see above). Therefore, *C. vidua* was a rather enigmatic species.

Cywinska & Hebert (2002) explained the high observed genetic variability, based on their allozyme and COI sequence divergences, by recurrent colonisation events combined with *in situ* mutational diversification. Now, *C. vidua* is shown to be a species with mixed reproduction including sexual and asexual females and males as mentioned above. At least in the Woods Hole population, these three genders co-exist in one pond, so that males have access to both sexual and asexual females. Further indications of the fact that males in this population are truly functional are the documented incidence of insemination in a sexual female by sperm, caught during preservation of the specimen (Fig. 6) and the discovery of copula (Fig. 7).

The existence of mixed populations in cypridid ostracods was abundantly demonstrated in *Eucypris virens* (see bode *et al*. 2010). This led researchers to postulate that occasional intraspecific hybridisations between males and asexual females in the same populations could give rise to polyploid asexual females (Adolfsson *et al*. 2009). This could be a plausible causal mechanism for the observed hypervariability in *E. virens*. The same could be true for *C. vidua* as indeed, polyploidy has been demonstrated for this species (Havel & Hebert 1989).

### Morphology of *C. vidua* with polychromatic polarization microscopy

The morphology of the prehensile palps and of the hemipenis in *Cypridopsis vidua* holds few surprises. The hemipenes are slightly asymmetrical, consist of ls and ms and internally the postlabyrinthal coils in the spermiduct show first the double coil, followed by an additional half coil leading to the basal sclerified part of the bursa copulatrix. The thumb-like expansions of the ls of the hemipenes are peculiar structures, not common in the Cyprididae, but also not unique in other Cypridopsinae. Here, however, these expansions are hyper-developed.

Especially in the illustrations of the polychromatic polarization microscopy of the hemipenes (Fig. 4A–B) the various parts of the pre-labyrinthal, labyrinthal and post-labyrinthal spermiduct are clearer than any other (non-stacking) photography could produce. This is important, as drawings give more detail, but these are always subjective interpretations, while the polychromatic polarization microscope photographs are fully objective pictures of the internal anatomy. This technique is non-invasive (it does not require chemical staining) and might be especially useful in re-analysing dissected older museum specimens.

The polychromatic polarization microscope photographs of the T1 of the male, showing the Rpp (Fig. 4C), are especially useful to illustrate the musculature of this (and other) limbs. Again, the shape of the second segment is shown objectively, while the drawing (Fig. 3B) is a subjective attempt to catch a three-dimensional structure in two dimensions. Also, the posterior part of the female body showing the insemination in progress (Fig. 6) has a clarity and detail which would be almost impossible to match in a drawing.

### Males in other *Cypridopsis* species

Of all the species listed by Meisch *et al*. (2019) in the genus *Cypridopsis*, many do not belong in this genus. At least 17 of them, described by Sars (1910) and Rome (1962) from Lake Tanganyika (Africa), belong in one or two new genera (Jacobs & Martens 2022). *Cypridopsis acanthodes* Rome, 1962 almost certainly belongs to the genus *Tanganyikacypridopsis* Martens, 1985 (see discussion in Martens 1985), while *Cypridopsis cunningtoni* Sars, 1910 was recently transferred to *Malawidopsis* by Jacobs & Martens (2022). Of the remainder of the species listed by Meisch *et al*. (2019) which can putatively be retained in *Cypridopsis s.s*., only four were described including the male morphology. Of these, *Cypridopsis brevisetosa* Klie, 1943 from Morocco (Africa) has very elongated valves, short natatory setae on A2, and Hp, Lpp and Rpp which are very different from those described here for *C. vidua* (Klie, 1943), to the extent that the assignment of this species to *Cypridopsis s.s*. can also be doubted. Three others, however, have very similar to almost identical morphologies to *C. vidua*.

*Cypridopsis okeechobei* Furtos, 1936 from Lake Okeechobee in central Florida (USA) has a Cp shape, ornamentation and colouration which is highly similar to that of *C. vidua*, especially taking into account that the latter species is known to have a highly variable Cp morphology (Meisch 2000). Furtos (1936), in addition, only cited small differences between her species and *C. vidua* in soft part morphology: small differences in the length of natatory setae on A2 and a shorter claw on T2, which may be part of intraspecific variability. But Ferguson (1964) also remarked that the natatory setae on the A2 in *C. okeechobei* extend beyond the tips of the claws by one-half the length of the claws, which is about the same as the in the illustration of the A2 of *C. vidua* in Meisch (2000: Fig. 156A). The main argument to distinguish *C. okeechobei* from *C. vidua* was apparently the absence of males in the latter species, and as was already foreshadowed in the introduction, we do not adhere to this particular species concept where sexual and asexual populations are lodged in different species. This had lead Martens & Savatenalinton (2011) to sink *C. okeechobei* into the synonymy of *C. vidua*, but at that stage based exclusively on the female (valve and carapace) morphology. Now we can also compare the male morphologies of *C. okeechobei* and *C. vidua*, and whereas the Hp (especially the large thumb-like expansion on the ls) and the Rpp are almost identical, the second segment of the Lpp is somewhat different between both species.

There is a lot of confusion in the literature regarding the identity of *Cypridopsis okeechobei*, possibly as a result of the way in which Furtos (1936) tried to distinguish her new species from *Cypridopsis vidua*. Delorme (1970) very briefly redescribed *C. okeechobei* and provided illustrations of a LV and a RV of a single individual. These figures show that these valves do not belong to a specimen of *C. okeechobei*, as the dorsal margin is much more rounded than in the figures of Furtos (1936). Delorme (1970) also remarked that *C. okeechobei* can be differentiated from *C. vidua* especially in being smaller in all dimensions, particularly height. But Furtos (1936: 492) explicitly stated that “This species may very readily be mistaken from *C. vidua vidua* by virtue of the pitted, hairy nature of the valve, presence of conspicuous dorso-lateral bands and similar size.”

Smith & Delorme (2010) provided different illustrations of what they call *Cypridopsis okeechobei* (and one of those figures is re-used by Smith & Horne 2016) but it is again doubtful if this is the real *C. okeechobei* as the LV is much more elongated (L / H ratio of this specimen = 1.90, L / H ratio of the illustration by Furtos (1936) = 1.58), while this specimen also has an anteroventral feature (widening of the fused zone) which does not occur in *C. vidua*). Several other reports on occurrences of *C. okeechobei* (e.g., Taylor & Howard 1993; Kaufmann *et al*. 2002; Fleury *et al*. 2014; Forester *et al*. 2017) do not provide illustrations or descriptions of what they call *C. okeechobei*, so if they based their work on any of the above three papers, then these (and other) identifications are uncertain. Our conclusion at this stage is that several populations have erroneously been identified as *C. okeechobei* and to go any further into the specific status of these populations is beyond the scope of the present paper.

*Cypridopsis howei* Ferguson, 1964 from Louisiana (USA) has a highly similar Hp morphology, also with the hyperdeveloped thumb-like expansion on the ls. Sadly, Ferguson (1964) did not illustrate or describe the prehensile palps. He did compare his species to *C. okeechobei*, citing differences in external valve ornamentation and shorter natatory setae on the A2 in his species, again characters that are prone to intraspecific variation. However, as the description and the illustration of *C. howei* is incomplete, and unless type material allows redescription, it might in time be better to consider *C. howei* an “uncertain species”, following the procedure of Meisch *et al*. (2019).

Cole (1965) described *Cypridopsis compressa*, later renamed *C. bisexualis* by Cole (1966), from Tennessee. From the illustrations, it is quite obvious that the valves of her specimens must have been severely decalcified, to the extent that the original shape was hardly imaginable. However, the shape of the Hp, including the large thumb-like expansion on the ls, and of the prehensile palps is very similar to that of *C. vidua*, as far as can be guessed from the illustrations. Cole (1965) did compare her species to *C. okeechobei* and to *C. howei* from which she distinguished it mainly by small differences in valve shape and ornamentation. Figure 39 in Cole (1965) shows that seta g on T2 is much longer than in *C. vidua*. As was suggested for *C. howei*, it might in time be better to classify *C. bisexualis* as an “uncertain species”.

Recently, Külköylüoglu *et al*. (2022) described *Cypridopsis schwartzi* from Texas. The shape and anatomy of the valves are very similar to those of *C. vidua*, while Hp and prehensile palps are most similar to those of *C. vidua* here described, although the second segment of the Lpp is somewhat more similar to that of *C. okeechobei*.

It is clear from the above analysis of various male morphological features, such as the hyperdeveloped thumb-like expansion on the ls of the Hp, that the latter four species are very closely related to *C. vidua*, and that most of the differences could be either owing to intraspecific variability (see extensive discussion in Meisch 2000: 387–388), or to artefacts related to illustrations. For example, the prehensile palps are often three-dimensional structures, and the way they are reduced to subjective two-dimensional drawings (when squeezed in a slide after dissection) can be different between specimens. In light of the present description of the males of *C. vidua*, it appears that most, if not all, of the above four species could be considered synonyms of *C. vidua*, but to confirm this is beyond the scope of the present paper.

Sexual (mixed) populations of *C. vidua* and related nominal species are thus far only known from North America, in spite of the fact that *C. vidua* is one of the most common non-marine ostracod species in the Holarctic and has also been found on all southern hemisphere continents, except Antarctica (Meisch *et al*. 2019). As was mentioned above, it is possible that other sexual populations have been overlooked, because it is difficult to identify the presence of males in this species without dissecting specimens. Also, in mixed populations, the ratio of males versus females (sexual and asexual) could be rather low. For example, Cushman (1907) already reported *Cypridopsis vidua* from south-eastern Massachusetts, where Woods Hole is situated, from “… all kinds of freshwater”. He only reported on finding female specimens, but maybe the elusive males had also escaped his attention?

## Conclusions

We here report on the first sexual (mixed) population of the common freshwater ostracod species *Cypridopsis vidua* from the northeast of the USA and describe the morphology of the male of this species. For the first time, we apply the polychromatic polarization microscopy technology for the illustrations of ostracod soft parts and find that this technique can clarify morphologies which otherwise remain less visible. We also analysed other species of *Cypridopsis s.s*. from which males were reported and find that at least four of them appear morphologically very closely related to *C. vidua*.

As males in this species are very difficult to identify without dissection, further populations of *C. vidua* from North America and from around the European and North African Mediterranean region (Horne & Martens 1999) should be screened carefully for the presence of males. This is important as this species is used as a test object in many biological disciplines, such as evolutionary biology, ecology, palaeo-ecology, toxicology and others. Whether populations of this species used for research in these various disciplines are fully asexual or have a sexual component can be highly relevant.

## Supplementary Material

1

## Figures and Tables

**TABLE 1:** Measurements (in μm) of selected specimens of *C. vidua* from the Woods Hole population by Scanning Electron Microscopy.

INV nrs	sex/asex	♂/♀	RV		LV		CpRL		CpD/V		L/W
			L	H	L	H	L	H	L	W	
INV.190338/1	asex	♀	–	–	–	–	–	–	634	457	1.39
INV.190338/2	asex	♀	–	–	–	–	648	425	–	–	–
INV.190338/3	asex	♀	–	–	–	–	–	–	652	463	1.41
INV.190340	asex	♀	–	–	–	–	–	–	627	426	1.47
INV.190339/1	sex	♀	–	–	–	–	–	–	590	411	1.44
INV.190339/2	sex	♀	–	–	–	–	581	397	–	–	–
INV.190341/1	sex	♂	–	–	–	–	–	–	558	407	1.37
INV.190341/2	sex	♂	–	–	–	–	543	363	–	–	–
INV.190341/3	sex	♂	–	–	–	–	–	–	575	387	1.49
INV.190341/4	sex	♂	–	–	–	–	–	–	563	373	1.51
INV.190336	sex	♂	564	363	572	362	–	–	–	–	–

## Abbreviations used in text and figures

Cp: Carapace
CpD/CpV: Carapace in dorsal/ ventral view
CpRL: Carapace in right lateral view
H: Height of valves
il: inner list
L: Length of valves
LV: Left valve
LVi: internal view of LV
RV: Right valve
RVi: internal view of RV
W: width of carapace
A1: antennula
A2: antenna
CR: caudal ramus (furca)
F: Female
FCO: Female Copulatory Organ
Hp: hemipenis
Lpp: left prehensile palp of T1 in male
ls: lateral shield of hemipenis
M: male
Md: mandibula
ms: medial shield of hemipenis
Mx1: maxillula
Rpp: right prehensile palp of T1 in male
T1: first thoracopod
T2: second thoracopod
T3: third thoracopod
