# Supplementary material for: The common morphospecies Cypridopsis vidua (O.F. Müller, 1776) (Crustacea, Ostracoda) is not an obligate parthenogen
Source: Belg J Zool. Author manuscript; Available in PMC 2025 Jul 30. (PMC12309733; doi:10.26496/bjz.2023.107)
Supplement: 1 [file NIHMS2035971-supplement-1.pdf]

Appendix

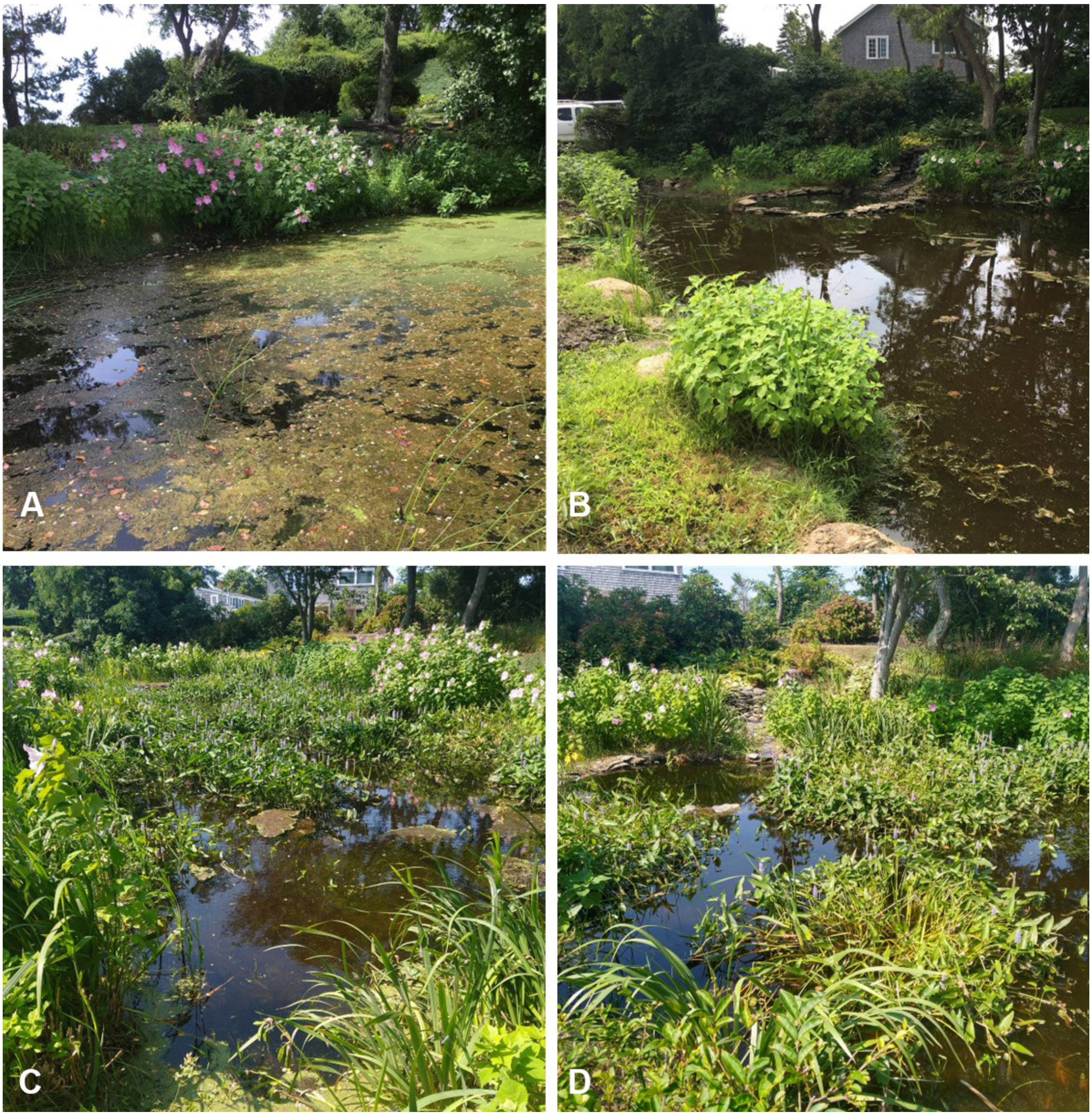

Figure S1 – Roadside pool on Gardiner Rd, Woods Hole, Ma (USA). **A**. Full of algal masses in 2019. **B**. After mechanical cleaning in 2019. **C–D**. Ecological changes in the pond in 2022.
